# Detection of Missing Bolts for Engineering Structures in Natural Environment Using Machine Vision and Deep Learning

**DOI:** 10.3390/s23125655

**Published:** 2023-06-16

**Authors:** Zhenglin Yang, Yadian Zhao, Chao Xu

**Affiliations:** 1 School of Astronautics, Northwestern Polytechnical University, Xi’an 710072, China; 963415148@mail.nwpu.edu.cn; 2 Taicang Yangtze River Delta Research Institute, Northwestern Polytechnical University, Suzhou 215400, China; yartinz@mail.nwpu.edu.cn

**Keywords:** object detection, deep learning, machine vision, bolt loosening, structural health monitoring

## Abstract

The development of an accurate and efficient method for detecting missing bolts in engineering structures is crucial. To this end, a missing bolt detection method that leveraged machine vision and deep learning was developed. First, a comprehensive dataset of bolt images captured under natural conditions was constructed, which improved the generality and recognition accuracy of the trained bolt target detection model. Second, three deep learning network models, namely, YOLOv4, YOLOv5s, and YOLOXs, were compared, and YOLOv5s was selected as the bolt target detection model. With YOLOv5s as the target recognition model, the bolt head and bolt nut had average precisions of 0.93 and 0.903, respectively. Third, a missing bolt detection method based on perspective transformation and IoU was presented and validated under laboratory conditions. Finally, the proposed method was applied to an actual footbridge structure to test its feasibility and effectiveness in real engineering scenarios. The experimental results showed that the proposed method could accurately identify bolt targets with a confidence level of over 80% and detect missing bolts under different image distances, perspective angles, light intensities, and image resolutions. Moreover, the experimental results on a footbridge demonstrated that the proposed method could reliably detect the missing bolt even at a shooting distance of 1 m. The proposed method provided a low-cost, efficient, and automated technical solution for the safety management of bolted connection components in engineering structures.

## 1. Introduction

Bolted joints are common and important in civil, mechanical, and aerospace engineering fields. On the other hand, bolt loosening is almost inevitable due to design, tight processing, load, and environment effects, which causes a very concerning problem for structural safety management [1]. For instance, in May 2020, the collapse of a construction hoist in Yulin, Guangxi Province, caused six fatalities due to two missing bolts at the top. Regular manual inspections are commonly used to ensure the safety of bolted connections. However, this approach is expensive, time-consuming, and reliant on the expertise of the inspector. Therefore, there is an urgent need to develop a technology that can accurately, rapidly, and automatically detect whether a bolt is missing or not in engineering.

In the past two decades, computer vision (CV) technology has emerged as a low-cost and efficient method to detect structural deformation [2,3] and damages [4,5]. Recently, CV technology has also been introduced for the aim of bolt loosening detection. Zhang et al. [6] proposed a novel structured light method based on motion image (SLMMI) for moving object inspection, which has been successfully used to inspect loose fasteners on high-speed railways. Zhou et al. [7] proposed an automated vision method for scanning angle taps to detect missing bolts during loading operations. Dou et al. [8] developed a Fast Template Matching (FTM) algorithm to identify the presence or absence of bolts. Similarly, Manikandan et al. [9] proposed a machine vision-based fully automatic detection and classification of a missing bolt detection system using a Support Vector Machine (SVM) classifier. These studies demonstrate the potential of computer vision in bolt loosening detection. In contrast to other bolt loosening detection methods, such as vibration or wave-based methods, the visual inspection is less susceptible to environmental factors such as temperature, humidity, and noise [10]. Furthermore, it can be utilized in complex engineering scenarios and has the capability to detect multiple loosened bolts simultaneously.

The visual inspection method involves the capture of images using various image sensors, such as digital cameras and mobile phones, which are subsequently processed to determine the degree of tightness of a target bolt. The current methods for visually inspecting bolt looseness can be classified into three categories. The first category involves measuring the extent of bolt looseness by determining the length of the exposed stud of the target bolt. Cha et al. [11] used features such as the horizontal and vertical lengths of bolt heads obtained from images to train a linear support vector machine to build a robust classifier that can automatically distinguish between tight and loose bolts. Ramana et al. [12] utilized the Viola–Jones algorithm to identify bolts and a trained support vector machine to classify tightened and loosened bolts. Sun et al. [13] used CNN to extract and segment the sub-pixel edges of the bolt cap and mounting surface before constructing 3D data of the boundaries based on stereo matching and binocular vision models to calculate the distance between the bolt cap and the mounting surface. Yuan et al. [14] proposed an automatic detection method using Mask RCNN, which marks defects for each pixel in the image to detect bolt loosening. Quantifying the degree of looseness by measuring the angle of looseness is the second category. Park et al. [15] employed the Hough circle transform to identify and segment the bolt image and then used Canny edge detection and the Hough transform to determine the rotation angle of the bolt. Liu et al. [16] instead used the affine transform to correct the image and then obtained the bolt contours by feature extraction and morphological processing. Huynh et al. [17] used the trained RCNN to identify the bolts and introduced the perspective transformation to correct the captured images. Wang et al. [18] introduced density-based noise based on prior studies and applied spatial clustering to enhance the accuracy of angle measurements based on the Hough transform results. Zhao et al. [19] measured the bolt rotation angle by determining the “num” on the bolt’s head instead of identifying the edge of the bolt. Pham et al. [20] suggested using bolted synthetic images generated from graphical models to train deep learning models. Yu et al. [21] calculated the bolt loosening angle by identifying the nut specification number and the markings made by the experiment. Sun et al. [22] introduced two different circular marks on the bolt and nut and then calculated the bolt rotation angle by identifying the circular marks. The third category is monitoring and quantifying bolt rotation using the target-free object tracking algorithm based on optical flow. Kong et al. [23] proposed an image-based feature tracking approach to locate loosening bolts. Pan et al. [24] continuously monitor and quantify the rotation of structural bolts using an optical flow-based target-free tracking algorithm after discovering the structural bolts by trained YOLOv3-tiny.

The previous research shows the potential of using vision techniques and deep learning to detect bolted targets in engineering structures. Data-driven deep learning approaches depend heavily on datasets, especially for target detection [25]. However, previous studies involving bolt identification sessions have produced datasets that are too idealized for real natural environmental conditions, limiting their generalizability for bolt identification. Zhao et al. [19] constructed a dataset with a single type of numbered bolt and divided it into two categories, “bolt” and “num”, to train the bolt identification and loosening detection models. Although they achieved a recognition accuracy of 0.914, the dataset used for training and testing had the same structure, which may not apply to other structures and types of bolts. Yang et al. [26] and Pham et al. [20] collected synthetic bolt datasets using 3D modeling tools to save time for collecting real-world training and test sets. However, they did not consider the effects of natural wear and corrosion on the bolts, and they did not account for the difference between the bolts and the background. Li et al. [27] generated images and videos from different locations of a steel truss bridge in Wuxi, Jiangsu Province, China, but the dataset environment was homogeneous, limiting the bolt recognition model’s generalizability. Yu et al. [21] and Sun et al. [22] acquired bolt images using smartphones at a distance of 8 to 10 cm. However, their trained models were based on a single dataset environment and had auxiliary markers. To enhance the bolt recognition models’ robustness and practicality, this study introduced a new dataset of bolt targets for computer vision and deep-learning-based bolt defect detection in engineering applications. The dataset mainly consisted of images captured from real-world structures under various natural conditions, while a small part was created using 3D modeling tools to augment the dataset. By incorporating the natural environments’ variability and complexity, this dataset enabled the development of more generalizable and effective bolt recognition models for real-world applications.

The following sections will introduce the proposed missing bolt detection framework and the constructed dataset. Then the detection principles based on the YOLO series of models and the evaluation metrics for the deep learning models’ detection results, as well as the specific missing bolt detection methods, will be described. Next, the performance of YOLOv4, YOLOv5s, and YOLOXs will be compared. Moreover, since YOLOv5s had the best detection results for bolt loosening detection, YOLOv5s was chosen to validate its bolt identification robustness under laboratory conditions and the missing bolt detection method’s robustness based on this model. Finally, the proposed method’s ability to detect missing bolts in real engineering cases will be verified.

## 2. NPU-BOLT Dataset

In the past, most deep-learning-based studies of bolt target recognition were based on images captured in the laboratory [21] and synthesized by 3D modeling software [20]. However, the two image acquisition methods above can capture bolts under only a few capture conditions. These bolts are usually structurally intact without damage and exposed without any concealment. In practical engineering, authentic bolt images generally have blurred edges, a particular perspective, and similar colors to the background, making recognition models trained by the above methods inaccurate or invalid. A dataset with realistic natural scene images called NPU-BOLT was developed in this study to make the bolt recognition model more practical.

To maximize the generalizability of the bolt recognition model, most of the bolt images in this dataset were acquired from natural scenes. Shadows can alter the brightness and contrast of objects, potentially making it challenging for the model to accurately discern object edges and contours; rust can modify the texture and shape of objects, potentially making it difficult for the model to recognize object features and categories; and surface color can alter the color distribution and reflectance of objects, making it difficult for the model to utilize color information for classification and regression purposes. To address these practical factors, this study accounts for the influence of shadows, rust, and bolt surface color during the acquisition process to ensure that training outcomes are not compromised.

Bolt images of the natural environment were captured with various devices, including a UAV and mobile phones. The specific characteristics of the sampling devices and the number of images captured by each device are shown in Table 1. In addition to photographs of bolts captured in natural scenes, the dataset also contained photos of bolts generated by three-dimensional modeling software and images downloaded from the internet. It is also worth noting that the dataset did not use any pictures of bolt structures in a laboratory environment. The final dataset contained 603 photos and a total of 2014 bolt targets.

**Figure 1:**
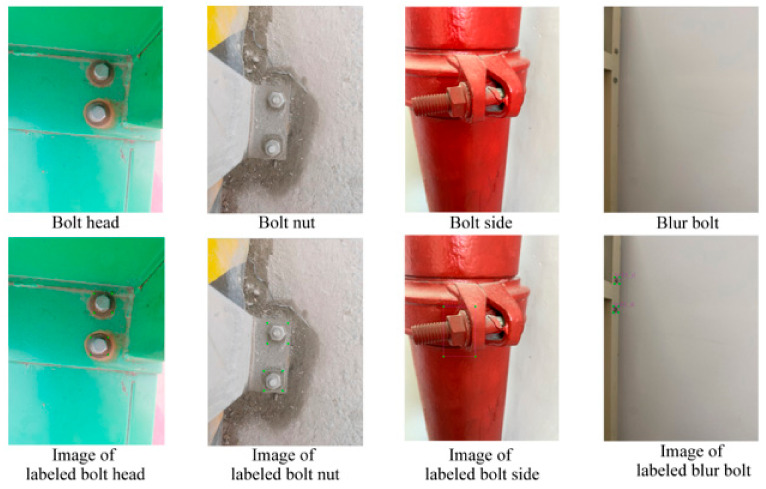
Four categories in bolt target detection and the corresponding labeled images.

**Figure 2:**
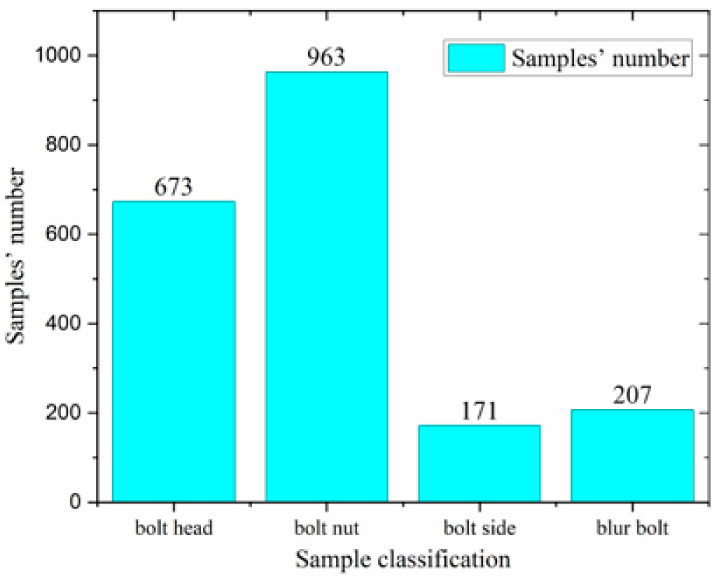
Number of categories of statistics.

To improve bolt identification accuracy, the bolt’s exposure and the shot’s angle were used to classify the bolt. Four classification labels were defined: “bolt head”, “bolt side”, “bolt nut”, and “blur bolt”. The bolt image was then manually labeled with LabelImg to retrieve the ground truth bounding box. Four categories in bolt target detection and the corresponding labeled images are illustrated in Figure 1, and the statistics for the four categories of classification are presented in Figure 2.

Finally, the developed dataset was uploaded to the site https://www.kaggle.com/datasets/yartinz/npu-bolt (accessed on 22 April 2023).

## 3. Deep Learning Model for Bolt Target Detection

After an image database was constructed, deep learning methods were first trained by these labeled images in the database. Upon evaluation of the recognition accuracy, the optimal model was selected for bolt recognition in accordance with the objectives of the present study. There are many deep learning methods for target detection, such as Faster R-CNN [28], YOLO-series algorithms, and SSD [29]. To date, several studies [30,31,32] have compared the performance of the YOLO series of algorithms with other real-time deep learning algorithms. From these studies, it is evident that among several commonly used deep-learning-based target detection algorithms, the YOLO series of algorithms typically achieve higher accuracy and speed.

**Figure 3:**
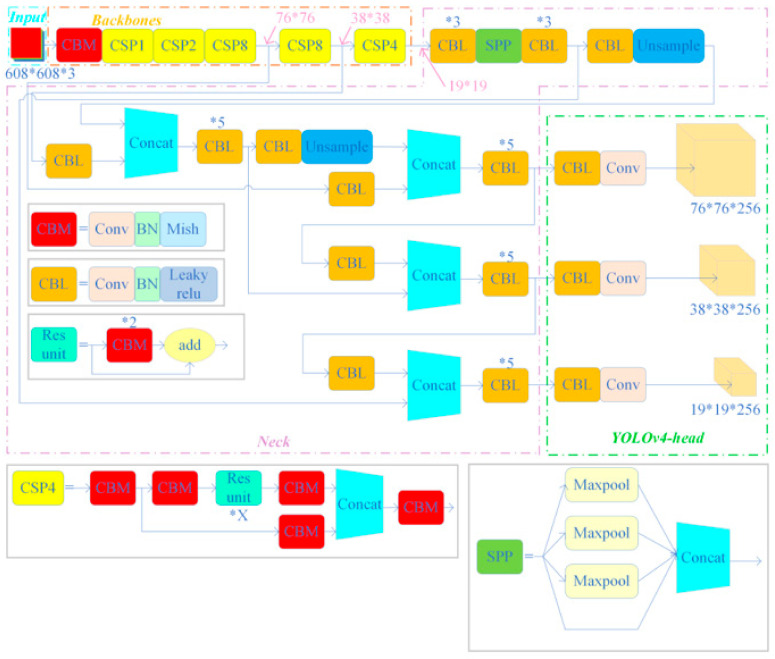
YOLOv4 network structure.

**Figure 4:**
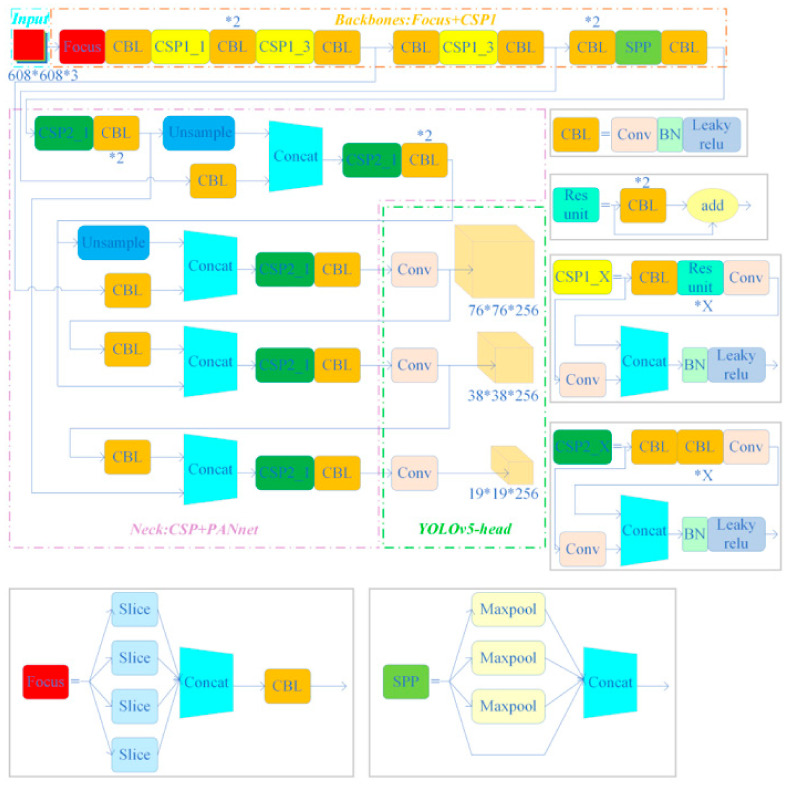
YOLOv5s network structure.

**Figure 5:**
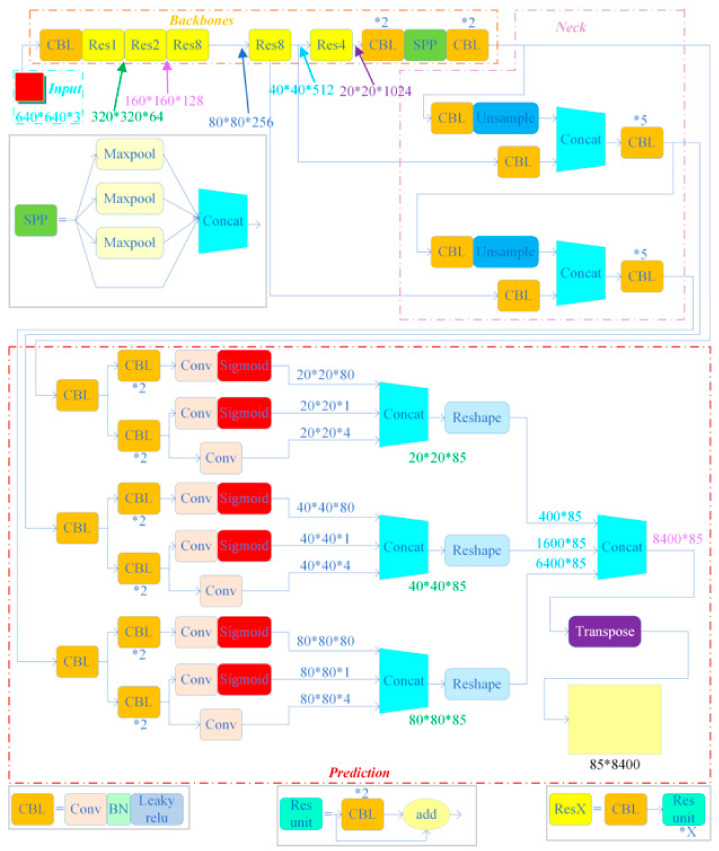
YOLOXs network structure.

In the context of engineering bolt identification and loosening detection, having a highly accurate and efficient identification model is crucial. Therefore, this paper adopted a deep learning model based on the YOLO series of algorithms to implement the proposed bolt target detection framework. Three good-performance algorithms used in other fields, namely, YOLOv4, YOLOv5, and YOLOX, were also compared. The three deep learning models of the YOLO series chosen for comparing recognition accuracy in this paper were all submitted after improving the YOLOv3 [33] -based deep learning model [32,34]. Since the three deep learning models were similar in structure, a brief description of the network structure of YOLOv4 and the main differences between the three models are shown below. In addition, the network structures of these three models are shown in Figure 3, Figure 4, and Figure 5, respectively.

### 3.1. YOLOv4 Network Architecture

The backbone network of YOLOv4 is based on a modified Darknet-53 model (CSPDarknet53), which is a deep convolutional neural network composed of 53 convolutional layers. The network includes five residual blocks, generating multiple feature maps that enable target detection at various scales. The features obtained from the backbone network are used to construct a feature pyramid with high resolution, which is achieved by leveraging the Spatial Pyramid Pooling (SPP) [35,36] structure and Path Aggregation Network (PANet) [36]. The SPP structure applies maximum pooling layers of different sizes to extract contextual information at different scales, while the PANet fuses features at different levels through lateral connections. YOLOv4 introduces three YOLO heads for classification and regression, which use anchor points of various sizes to accommodate target regions of different scales. The YOLO head takes the feature map as input, utilizing a 1 × 1 convolutional layer and a 3 × 3 convolutional layer to predict the class and bounding box of each anchor point. Finally, YOLOv4 employs several optimization techniques, such as batch normalization (BN), a Mish activation layer [37], and cross-stage section (CSP), to enhance its performance.

### 3.2. The Main Differences between the Three Models

The YOLOv4, YOLOv5s, and YOLOXs architectures differ mainly in their backbone networks. Specifically, YOLOv4 employs the CSPDarknet53 as the backbone network, which incorporates modules such as SPP, PAN, and SAM to enhance feature extraction and fusion. Meanwhile, YOLOv5s adopts a lightweight backbone network called FocusNet, which utilizes a Focus module to downscale the input resolution and reduce the computational burden. Similarly, YOLOXs also uses FocusNet as the backbone network but integrates a ResNet-like structure, named YOLOPAFPN, in the Neck section, which uses a Path Aggregation module to realize top-down and bottom-up feature fusion. Additionally, YOLOv4 adheres to the anchor frame settings of YOLOv3, which entails obtaining nine pre-defined anchor frames through clustering analysis and assigning them to three different scales of detection layers. In contrast, YOLOv5s employs an adaptive anchor frame setting that automatically learns the optimal anchor frame size and scale based on the training dataset. Likewise, YOLOX also utilizes an adaptive anchor frame setting but reduces the number of anchor frames from nine to six to minimize overlap and redundancy.

### 3.3. Evaluation Metrics

The precision, recall, mean average precision (mAP), and *F*_β_ score are given in this paper to verify the validity and accuracy of the model, and the mathematical functions of them are given as follows:

(1) $$Precision=\frac{TP}{TP+FP}$$

where *TP* is the true positive, and *FP* is the false positive.

(2) $$Recall=\frac{TP}{TP+FN}$$

where *FN* is the false negative.

(3) $$Average\;precision=\int_{0}^{1}Precision(recall)d(recall)$$

(4) $$mAP=\frac{\sum_{N}^{n=1}AP(n)}{N}$$

where *n* is the number of categories, and *N* is the total number of classes.

(5) $$F_{\beta }=\frac{(1+\beta^{2})\times P\times R}{(\beta^{2}\times P)+R}$$

The relative importance of recall on precision is greater than 0, where *β* > 1 affects recall more, and *β* < 1 affects precision more. Since the model detected missing bolts by comparing the difference between the number of bolts in the engineering plans and the number of identified bolts, the false detection of non-bolted objects as bolts made the number of identified bolts high and the number of missing bolts low, leading to safety hazards, so false detection should be avoided as much as possible. Therefore, the precision rate is more important. So let *β* = 0.5, which means an *F*_0.5_ score measures the model’s accuracy.

## 4. Missing Bolt Detection from Images

**Figure 6:**
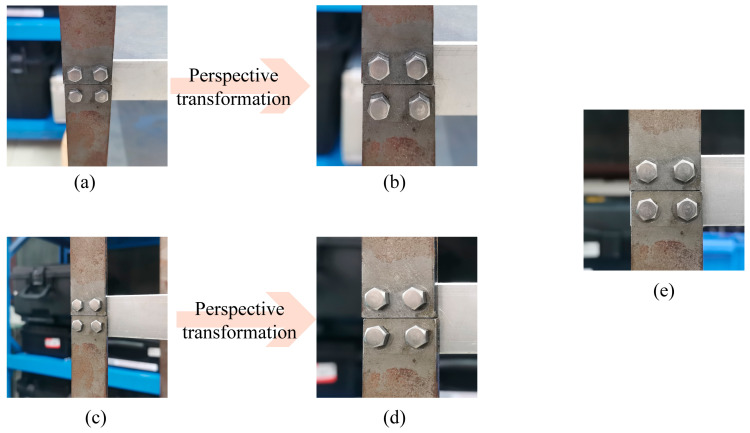
Perspective transformation with 30° tilt angle for bolt image reconstruction. (**a**–**d**) 30° tilt in the horizontal and vertical directions and transformed images, (**e**) reference image.

A reference image with a small perspective of the bolts was provided as the input to detect missing bolts, ensuring that no bolts were absent from the structure. Since the bolt reference image is an orthographic image and the inspection target image may have a specific perspective angle, the direct comparison of the target image with the reference image may cause errors. Thus, it was essential to correct the perspective of the inspection target image through perspective transform [38]. The perspective transform algorithm can rectify an image by mapping it onto a reconstructed view plane. The conversion formulae commonly used for the perspective transform are as follows:

(6) $$\left[\begin{array}{c} x^{\prime }\\ y^{\prime }\\ w^{\prime } \end{array}\right]=\left[\begin{array}{ccc} h_{11} & h_{12} & h_{13}\\ h_{21} & h_{22} & h_{23}\\ h_{31} & h_{32} & h_{33} \end{array}\right]\left[\begin{array}{c} u\\ v\\ w \end{array}\right]$$

(7) $$\left\{\begin{array}{c} x=\frac{x^{\prime }}{w^{\prime }}\\ y=\frac{y^{\prime }}{w^{\prime }} \end{array}\right.$$

where *u*, *v* represent the coordinates of the original image, and *x*, *y* represent the coordinates of the image after the perspective transformation. Prior knowledge of the coordinates of the four points of the image to be transformed and the corresponding coordinates of the four vertices of the transformed image rectangle were necessary for the successful implementation of the algorithm. This study developed an application that allowed the user to obtain the four points of the image to be transformed by clicking on the four vertices in the image, while the four vertices of the transformed image rectangle could be predetermined based on the real dimensions of the bolted constructions. The effectiveness of the perspective transformation was illustrated with an example of a 30° tilt in the horizontal and vertical directions, as shown in Figure 6.

**Figure 7:**
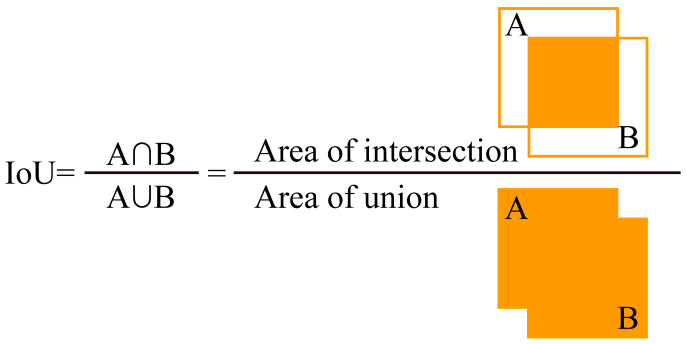
Intersection over union (IoU).

To ascertain whether a bolt was missing, the bolt identification result of the target image was compared with that of the reference image. The intersection over union (*IoU*) is a metric used to quantify the degree of overlap between two bounding boxes, by measuring the ratio of their intersection region to the merged region, as depicted in Figure 7. In the field of target recognition neural networks, *IoU* is typically used in the loss function for bounding box prediction [39]. The output of the YOLO models is a tensor containing predicted bounding boxes, confidence levels, and categories, where the bounding box comprises *x*, *y*, *w*, *h*, and the confidence score. Specifically, *x*, *y*, *w*, and h denote the center coordinates, width, and height of the bounding box, respectively. To determine if a bolt was missing in the target image, one only needed to calculate the *IoU* between each target bounding box in the reference image and each target bounding box in the detection target image.

Specifically, the intersection and union ratio (*IoU_ij_*) between the *i*th target bounding box in the reference image and the *j*th target bounding box in the detection target image was calculated and expressed by the following equation:

(8) $$IoU_{ij}=\frac{R_{i}\cap T_{j}}{R_{i}\cup T_{j}}$$

where *R_i_* denotes the *i*th target bounding box in the reference image and *T_j_* denotes the *j*th target bounding box in the detection target image. The missing index (*M_i_*) of the *i*th target bounding box in the reference image was then calculated and compared with the defined threshold. *M_i_* is the sum of the squares of the intersection and union ratios of the *i*th target bounding box in the reference image and all target bounding boxes in the detection target image, expressed by the following equation:

(9) $$M_{i}=\sum_{j=1}^{n}IoU_{ij}^{2}$$

where *n* is the number of bounding boxes in the detection target image. It could be deduced that if the *j*th bolt in the detection target image was not missing, then there must have been at least one bounding box of the *j*th bolt in the corresponding reference image with a non-zero intersection and union ratio, indicating that the missing index was not zero. Conversely, if the *j*th bolt in the detection target image was missing, the bounding box of the *j*th bolt in the corresponding reference image had a zero intersection and union ratio with all bounding boxes in the target image, indicating that the missing index was zero. Therefore, the value of *Thld* was simply set to 0 as follows:

(10) Thld=0

(11) $$M_{i}>Thld$$

The *i*th bolt was considered as not missing if the condition in Equation (11) was satisfied; otherwise, the bolt was missing. However, this criterion applied only if the detection image had a similar angle to the reference image and the bolt coordinates in the detection image were almost identical to those in the reference image. To detect missing bolts from a wider range of shooting angles, a perspective transformation was introduced before missing bolt detection. To minimize constraints on the bolt coordinates in captured images, relative center coordinates were used for all calculations related to *IoU* in this study. These coordinates represented the bounding box center with respect to a known rectangle in the bolt’s background, which could be either the rectangle that enclosed the bolt in the reference image or the four vertices forming a rectangle that were easily identified by the user. It should be noted that in the target detection image, the rectangle may become a quadrilateral due to the capture angle. Moreover, the user was responsible for determining the rectangle in the reference image and its corresponding quadrilateral in the target detection image in advance. The association between the rectangles for perspective transformation in the reference and detection images was leveraged. Part 5.3.5 will illustrate the impact of the perspective transformation with relative center coordinates.

## 5. Framework Flowchart

**Figure 8:**
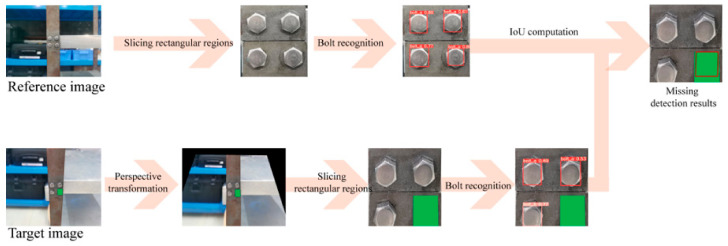
Flowchart of the developed framework.

Figure 8 illustrates the proposed framework for missing bolt detection, which followed the procedure described in Section 4. The reference image was first segmented into rectangular regions containing bolts, which were used to compute their relative center coordinates. Next, a deep learning neural network detected the bolts and extracted their target recognition boxes from the reference image, which were stored in the system. To detect a missing bolt in a test image, the test image underwent perspective transformation and rectangle segmentation. The same neural network detected and outputted the target recognition boxes of the bolts in the test image. Finally, by applying the IoU calculation method presented in Section 4, any missing bolts could be identified. The developed system was highly automated and only required users to click on the four corners of each rectangular region containing bolts in both the reference and test images.

## 6. Experimental Results and Discussion

### 6.1. Model Training

At the outset, three YOLO model variants were initially trained using the VOC 2007 dataset that contained images of over 20 object categories, and the learned weights of each deep learning model were utilized as the starting point for the retraining process. The retraining was performed on the NPU-Bolt dataset to attain accurate recognition of bolts. Despite the variation in input size, batch size, and other training parameters of the different YOLO model versions, the hyperparameters were kept unchanged to enable a direct comparison among the models, namely, YOLOv4, YOLOv5s, and YOLOXs. Considering the small dimensions of the bolts, the network input size was set to 640 × 640 pixels. The training parameters of the different models are illustrated in Table 2. The environment configuration parameters for model training and testing are shown in Table 3.

### 6.2. Comparison of Training Results for Different YOLO Models

**Figure 9:**
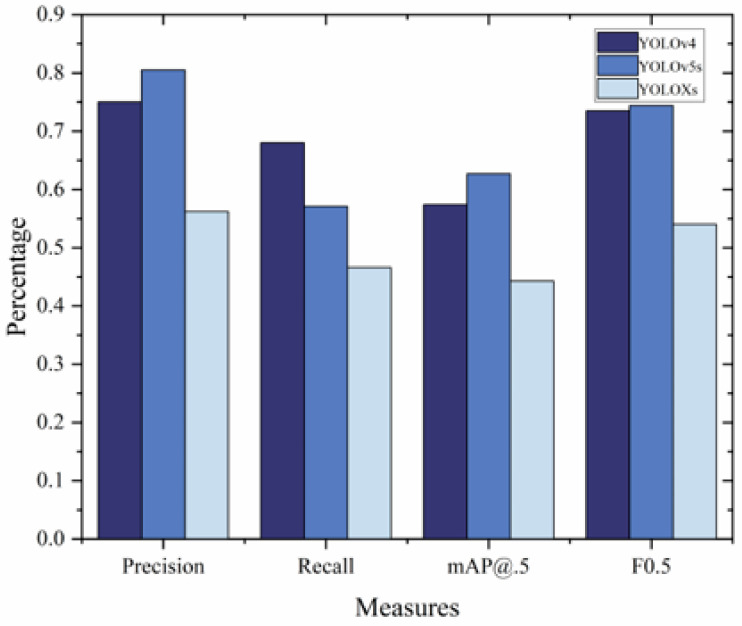
Results of comparing YOLOv4, YOLOv5s, and YOLOXs algorithms for bolt detection.

To ensure a fair comparison, all models based on the YOLO architecture were trained and tested on the identical training and test datasets divided by the NPU-Bolt dataset. The comparative results of bolt recognition for the three different algorithms trained using the NPU-Bolt dataset are shown in Figure 9. Compared with YOLOv4 and YOLOXs, YOLOv5s had a higher mAP, indicating that YOLOv5s could identify bolts more accurately than the other two algorithms after training on the NPU-Bolt dataset. YOLOv5s had higher precision than YOLOv4 due to the adaptive image scaling algorithm that improved the model’s overall precision.

**Figure 10:**
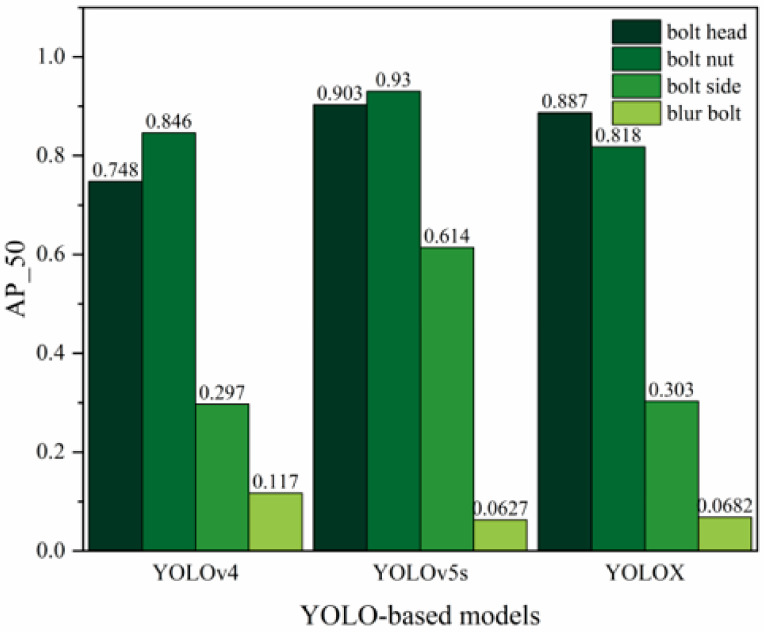
Performance of YOLOv4, YOLOv5s, and YOLOXs.

The recognition results of different networks for different bolt image classes are shown in Figure 10, which shows the average precision results of the three YOLO algorithms for all labels. It is worth noting that the category “blur bolt” included both blur nuts, blur bolt sides, and heads, with “blur” being a relatively subjective concept. Therefore, when the model accuracy was high, the model would likely identify the actual category of these vague bolts rather than the “blur bolts”. This phenomenon led to lower recall of “blur bolts” and hence a lower average accuracy for the “blur bolts” category.

### 6.3. Experiments under Different Laboratory Conditions

**Figure 11:**
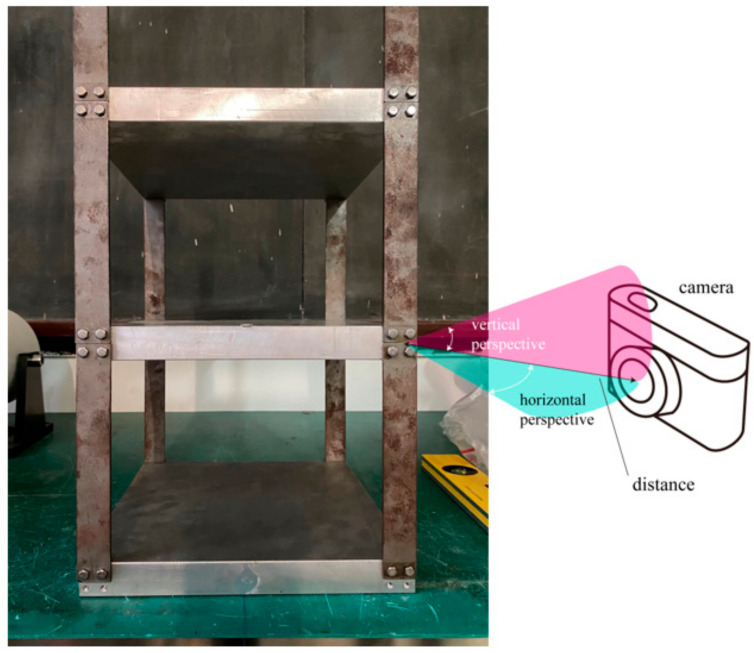
Experimental structure.

Section 4 explains that the proposed missing bolt detection method compared the target bounding box information of the detected target image and the reference image. If the target image and the reference image had the same angle and bolt coordinates, the detection accuracy of the missing bolt depended only on the bolt identification accuracy. Thus, this section first examines the bolt recognition capability of the deep learning model under different conditions. Then it verifies the effectiveness of the missing bolt detection method when the target image and the reference image have different angles and bolt coordinates. A bolted multi-story steel frame structure was constructed to test the model’s bolt identification capability and the method’s bolt absence detection capability. Figure 11 shows that the steel frame structure consisted of three layers of steel plates (250 mm × 250 mm × 25 mm), ten connecting steel pieces (175 × 25 × 10 mm), and several M4 hexagonal bolts. The front view showed 20 bolts. A camera (model: Canon 200D, parameters in Table 1) captured images of the bolt with a resolution of 3648 × 2736. YOLOv5s was chosen for experimental validation under different conditions based on its higher mAP. The study focused on verifying the model’s robustness for bolt identification detection under different environmental conditions and the effectiveness of the model-based missing bolt detection method. Images of the test samples were obtained by varying the shooting conditions, such as horizontal and vertical perspective angles, shooting distances, light intensities, and image resolutions. The test results will be analyzed in the following sections.

#### 6.3.1. Effect of Different Perspectives

**Figure 12:**
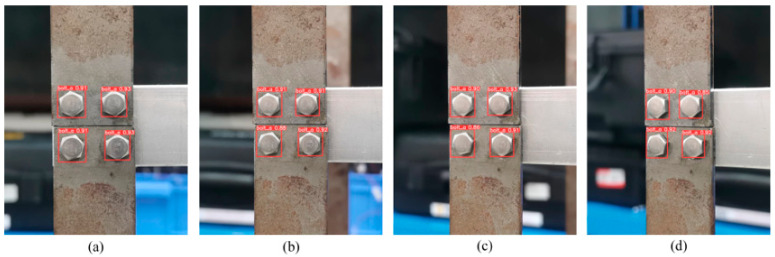
Detection results from different perspectives horizontally: (**a**) 0°, (**b**) 10°, (**c**) 20°, and (**d**) 30°.

**Figure 13:**
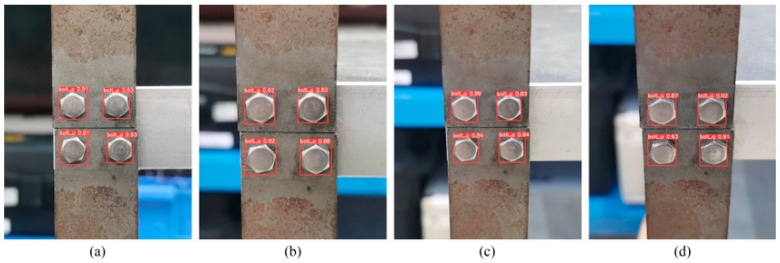
Detection results from different perspectives vertically: (**a**) 0°, (**b**) 10°, (**c**) 20°, and (**d**) 30°.

The robustness of the YOLOv5s model at different perspective angles was evaluated by taking sample images at horizontal angles (0°, 10°, 20°, 30°) and vertical angles (0°, 10°, 20°, 30°), as shown in Figure 12 and Figure 13. Bolts were accurately identified by the model at 30° or lower angles. However, the confidence level in identifying the target frame decreased as the perspective angle increased, indicating a lower accuracy. Furthermore, the confidence level for recognition at certain horizontal perspective angles was above 85%, but it was only above 80% at the same vertical perspective angles. Thus, the recognition accuracy of the model was more significantly decreased by increasing the vertical angle than by increasing the horizontal angle. Therefore, images at large perspective angles, especially vertical ones, should be avoided.

#### 6.3.2. Effect of Different Shooting Distances

**Figure 14:**
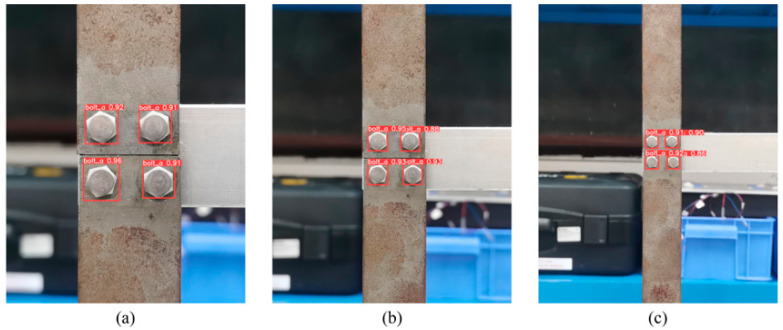
The detection results with different distance settings: (**a**) near, (**b**) medium, and (**c**) far.

The shooting distance was also a crucial factor. Therefore, this study used images taken at three different distances for validation. These were 15 cm for close range, 25 cm for medium distance, and 40 cm for far distance. As seen in Figure 14, the model accurately identified the bolts at all three different distances, and the confidence level fluctuated in almost the same range, which meant that the detection accuracy was not affected by the shooting distance of at least 40 cm.

#### 6.3.3. Effect of Different Light Conditions

**Figure 15:**
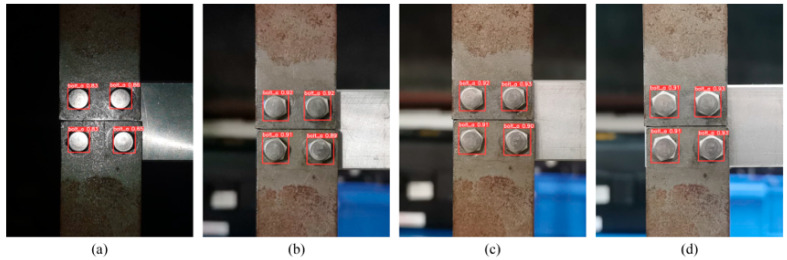
Detection results with different lighting conditions: (**a**) dark with flash on, (**b**) dim, (**c**) normal, and (**d**) bright.

The lighting environment created shadows, which could also affect the quality and sharpness of the image. Therefore, in this section, the effect of the lighting conditions on the model’s accuracy was tested by taking photos of the test sample under four lighting conditions: dark conditions with the flash on, dim, standard lighting, and fluorescent light on. As shown in Figure 15, the model could still accurately identify all bolts under the four lighting conditions above. However, it should also be noted that the confidence level in recognizing the target frame dropped significantly for the test sample images taken in dark conditions with the flash on. The results of this test suggested that lighting conditions had a more significant effect on recognition than the previous two influencing factors, so it is recommended that high-quality photographs in standard indoor lighting be used for recognition testing in practice.

#### 6.3.4. Effect of Different Image Resolutions

**Figure 16:**
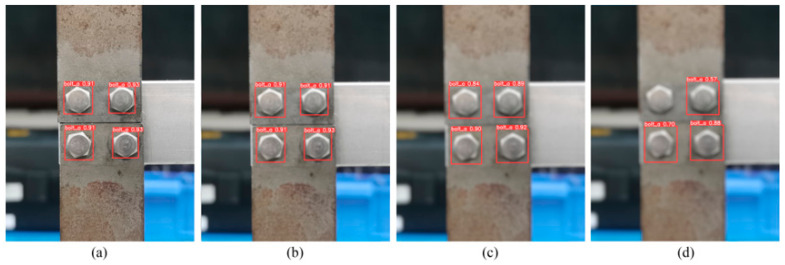
Results with different image qualities: (**a**) lens blur 0, (**b**) lens blur 10, (**c**) lens blur 20, and (**d**) lens blur 25.

UAVs are usually used to capture images for identifying missing bolts in real-world engineering structures with the proposed detection method in this study. However, UAVs tend to vibrate during flight, leading to poor image quality. To address this issue, four images with varying resolutions were captured with a hexagonal aperture and lens blur parameters of 0, 10, 20, and 25, where the latter parameter reflected the aperture radius and the blur degree. Figure 16 shows that the frame confidence level decreased with increasing blur and the YOLOv5s identified the bolt accurately when the lens blur was 0–20 but missed a detection at a lens blur of 25. This outcome could inform the minimum photo quality required for bolt loosening detection. However, it is worth noting that at a lens blur of 20, the photo quality was already below that of most surveillance cameras.

#### 6.3.5. Missing Bolt Detection for the Steel-Framed Structure

**Figure 17:**
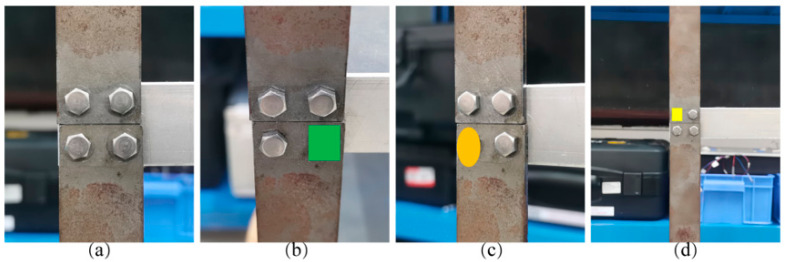
Reference and target images: (**a**) reference image, (**b**) target image from 30° vertical perspective, (**c**) target image from 30° horizontal angle, and (**d**) target image at 45 cm distance.

**Figure 18:**
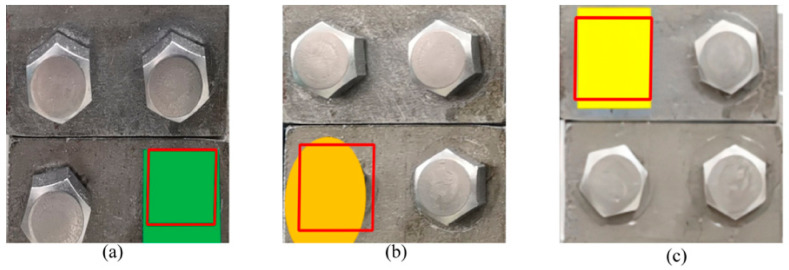
Results of missing detection with different conditions: (**a**) target image from 30° vertical angle, (**b**) target image from 30° horizontal angle, and (**c**) target image at 45 cm distance.

The proposed method was tested on target images with different shooting angles and distances while keeping the reference image to assess its practicality in real-world applications. Different shooting angles tested the impact of perspective transformation on missing bolt detection, and different distances tested the effect of relative center coordinates. The reference image and part of the target image, colored to simulate a bolt defect, are shown in Figure 17. The model identified the cropped rectangular image directly after the perspective transformation to eliminate the influence of bolt coordinates on the missing detection results since relative center coordinates depended on a rectangle or four vertices forming a rectangle in the image. Figure 18 shows the results, indicating that the perspective angle was accurate for missing detection up to 30°. Moreover, the bolt coordinates did not affect the missing detection outcomes.

### 6.4. Missing Bolt Detection for a Real-Scale Footbridge

#### 6.4.1. Experiment on a Footbridge

**Figure 19:**
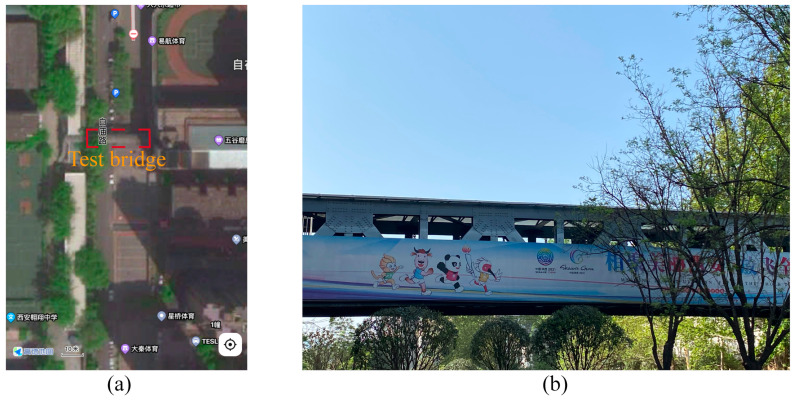
Pedestrian bridge for testing. (**a**) Bridge location (from Amap), (**b**) Real view of the bridge.

**Figure 20:**
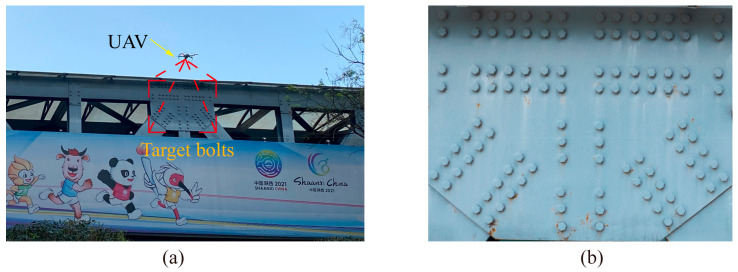
Image capture of bolted joints by UAV. (**a**) UAV approaching bolted connection and (**b**) target bolts.

The efficacy of the proposed missing bolt detection model was evaluated in a real-life bolted connection scenario. The test was conducted on a footbridge located in Xi’an, Shaanxi Province, China, as illustrated in Figure 19a. The footbridge consisted of numerous bolts, as displayed in Figure 19b. A specific bolted structure on the footbridge was selected as the test subject, as illustrated in Figure 20. Given the challenging accessibility of the test connection, the images of the bolts were obtained using a UAV.

The DJI Air2S drone was employed to obtain images of the bolt connections at a high resolution of 5464 × 3640 pixels, and its specifications are listed in Table 1. To capture the images, the drone was gradually steered toward the bolt connection under scrutiny, until it was situated approximately 1 m in front of it, as shown in Figure 20a. A cropped image of the target bolt was then taken with minimal perspective distortion (i.e., perspective angle ≤ 5°), which served as the reference image for the subsequent bolt defect detection. Furthermore, since the images were taken on a clear day, the shadows on the bridge were more prominent and provided a means of testing the model’s ability to identify defects under different lighting conditions. The cropped image of the target bolt is depicted in Figure 20b.

#### 6.4.2. Bolt Identification and Missing Bolt Detection Results

**Figure 21:**
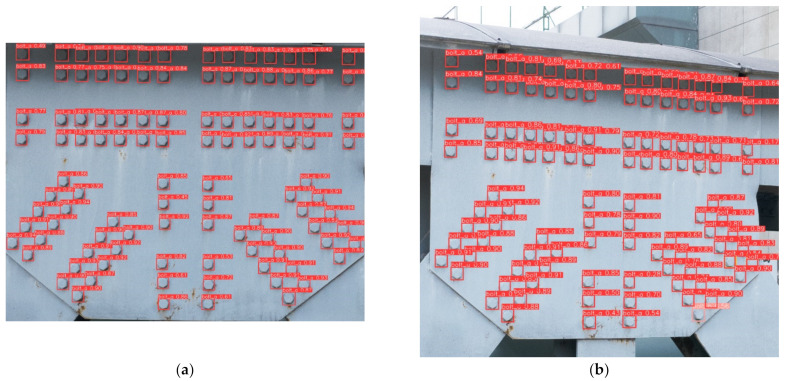
Bolt identification results: (**a**) small perspective (perspective angle ≤ 5°) angle bolt recognition and (**b**) perspective bolt identification.

**Figure 22:**
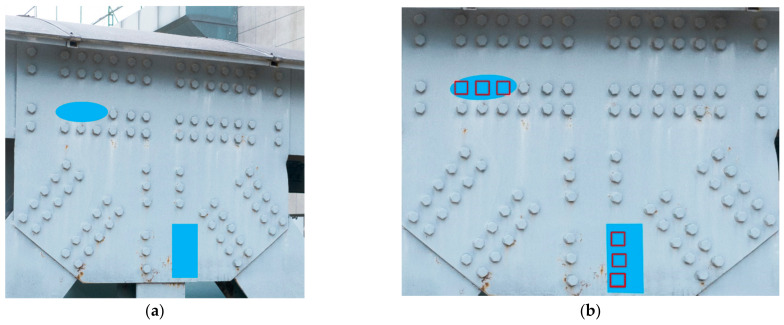
Detection of target images with perspective and missing detection results. (**a**) target images with perspective, (**b**) missing detection results.

The bolt identification results of the proposed method for a reference image and a test object taken at a certain perspective are presented in Figure 21. As depicted in Figure 21a, the model could accurately identify the bolt and the type of bolt (bolt head) when the test object was photographed at a small perspective, despite the differences in lighting conditions. However, when the test object was photographed from a certain perspective, as in Figure 21b, the model still recognized the bolt but misclassified the type of bolt. In the natural scene, bolts and nuts contained more intricate features, such as bolts, threads, and screw holes with threads, leading to the misclassification of a bolt head as a nut. The missing bolt detection results are shown in Figure 22, where the missing bolt is approximated by the overlaid geometry. These results demonstrated the capability of the proposed method to perform missing bolt detection in real engineering structures.

## 7. Conclusions

This paper proposed a novel approach for detecting missing bolts in bolted connections using deep learning and image processing techniques. The approach trained a bolt recognition model using a natural environment bolt dataset (NPU-Bolt) for automatic bolt detection in images. It also developed an image processing algorithm for detecting bolt absences based on perspective transformation and comparison of the relative center coordinates of the bolt recognition bounding box. Experiments on laboratory-scale bolted joints under varying conditions such as perspective distortion, shooting distance, image resolution, and light intensity validated the method’s effectiveness. The method was also tested on a real-world engineering structure, i.e., a footbridge connection, with numerous bolts. The experiments led to the following conclusions:

1. The model trained with the natural bolt dataset showed strong robustness and met the engineering application accuracy by validating bolt images obtained under different environmental conditions.
2. The proposed method detected missing bolts at arbitrary viewing angles robustly in laboratory tests. It detected bolt absences accurately at perspective angles ≤30°. Moreover, the model identified bolts accurately when the lens ambiguity was ≤20. However, the recognition accuracy decreased significantly when the lens ambiguity was >25. Thus, to ensure accurate recognition results, the image resolution (lens blur ≤ 20) should be increased as much as possible.
3. The field test results confirmed the proposed method’s potential for missing bolt detection in large bolted joints. The method performed consistently well in bolt defect detection and could be integrated with UAVs for quasi-real-time monitoring.

## Figures and Tables

**Table 1:** Specific parameters of the sampling device.

Model	Camera Sensor	Aperture	Focal Length	Image Resolution	Image Count
Canon 200D	24 MP	f/4.5	18–55 mm	6000 × 4000	21
DJI Air 2S	20 MP	f/2.8	22 mm	5472 × 3648	83
HonorX10	40 MP	f/1.8	38–24 mm	2736 × 3648	167
iPhone 11	12 MP	f/1.8	26 mm	4032 × 3024	135

**Table 2:** The training parameters of YOLO-based models.

Parameters	YOLOv4	YOLOv5s	YOLOXs
Input size	640 × 640	640 × 640	640 × 640
Learning rate	0.001	0.001	0.001
Momentum	0.949	0.937	0.900
Batch size	64	32	16
Number of categories	4	4	4
Epochs	300	300	300

**Table 3:** Environment configuration.

GPU	RTX 3090
System	UBUNTU 18.04
Pytorch version	1.7.1
CUDA version	11.0

## Data Availability

The dataset presented in this study are available in the site https://www.kaggle.com/datasets/yartinz/npu-bolt (accessed on 22 April 2023).
